# Evaluating anger in Chinese adolescents: Psychometric validation and clinical sensitivity of the Chinese version of the Children's Inventory of Anger

**DOI:** 10.1002/ped4.70024

**Published:** 2025-10-11

**Authors:** Xinyue Zhang, Liping Ma, Chi‐son Kuan, Xinli Chi, Yuan Cao, Suhong Wang, Chao Yan, W. Michael Nelson

**Affiliations:** ^1^ Key Laboratory of Brain Functional Genomics (MOE&STCSM) Affiliated Mental Health Center (ECNU) School of Psychology and Cognitive Science East China Normal University Shanghai China; ^2^ Shanghai Changning Mental Health Center Shanghai China; ^3^ School of Psychology Shen Zhen University Shenzhen Guangdong China; ^4^ Department of Social Work and Social Administration The University of Hong Kong Hong Kong China; ^5^ The First People's Hospital of Changzhou Changzhou Jiangsu China; ^6^ Key Laboratory of Philosophy and Social Science of Anhui Province on Adolescent Mental Health and Crisis Intelligence Intervention Hefei Anhui China; ^7^ Department of Psychology Xavier University Cincinnati Ohio USA

**Keywords:** Children's inventory of anger, Chinese, Clinical application, Measurement invariance, Reliability, Validity

## Abstract

**Importance:**

Accurately assessing anger in Chinese adolescents is crucial. Uncontrolled anger among adolescents can manifest in externalizing and internalizing problems. However, there are limited assessments specifically designed to measure anger in Chinese adolescents.

**Objective:**

This study aimed to evaluate the psychometric properties of the Chinese version of the Children's Inventory of Anger (ChIA‐C).

**Methods:**

We enrolled 1790 adolescents aged 11–18 years from five middle schools (mean age of 14.56 ± 1.71 years, 57.9% female). Of these, 901 participants (mean age of 13.95 ± 1.66 years, 59.2% female) completed a retest survey after 9 months. Psychometric assessments comprised factor structure analysis, reliability testing, validity examination, and measurement invariance analysis. Furthermore, we recruited 50 pairs of depressed adolescents and healthy controls to evaluate the clinical sensitivity of the ChIA‐C.

**Results:**

Factor analysis confirmed a robust four‐factor structure with favorable fit indices (*χ^2^/df* = 3.35, Root Mean Square Error of Approximation = 0.05, Comparative Fit Index = 0.92, and Tucker‐Lewis Index = 0.91). Internal consistency reliability (Cronbach's α = 0.76–0.92) and test‐retest reliability (*r* = 0.38–0.50, *P* < 0.001) were satisfactory. Positive correlations between ChIA‐C scores and those of the Anger Expression Scale for Children (*r* = 0.28–0.49, *P* < 0.001) supported moderate convergent validity. Concurrent and predictive validity were confirmed by significant positive correlations between ChIA‐C and Children's Depression Inventory scores at wave one and wave two, respectively (*r* = 0.13–0.34, *P* < 0.001). Moreover, measurement invariance across age and partial measurement invariance across sex were supported. Depressed adolescents showed significantly higher scores than healthy controls in the ChIA‐C and its authority relationship and physical aggression subscales.

**Interpretation:**

The ChIA‐C exhibits robust psychometric properties, establishing its reliability and validity as a tool for assessing anger in Chinese adolescents.

## Graphical abstract

The Chinese version of the Children's Inventory of Anger demonstrates strong reliability, validity, and a stable four‐factor structure. It effectively distinguishes anger levels in depressed versus healthy adolescents, highlighting its clinical utility for assessing anger‐related challenges in Chinese youth.

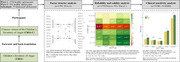

## INTRODUCTION

Anger is commonly characterized as a subjective emotional state encompassing irritation and heightened arousal of the autonomic nervous system.^1^, ^2^, ^3^ It plays a pivotal role in adolescents’ socioemotional development during critical transitions in learning, socialization, and relationship formation.^4^, ^5^ Heightened stress during these developmental stages frequently precipitates anger episodes,^6^ with maladaptive regulation leading to dual manifestations of externalizing and internalizing problems. Particularly, anger in adolescents is associated with a heightened risk of physical and verbal aggression, as well as other forms of indirect aggression,^2^, ^7^, ^8^ which are among the most prevalent and destructive behaviors in adolescents.^9^ Its significance extends to psychopathology, with anger playing a role in various disorders such as anxiety and depression,^10^, ^11^, ^12^ associated with suicidal ideation^13^ and persistent psychopathology.^14^ Thus, studying anger in children and adolescents is crucial for implementing effective anger management strategies and developing adaptive interventions.

The Children's Inventory of Anger (ChIA), a 39‐item scale assessing anger through four domains (frustration [FRUST], physical aggression [PHYS], peer relationship [PEER], and authority relations[AUTH]) in 6‐ to 16‐year‐olds,^15^ has gained recognition for its effectiveness in evaluating anger, as evidenced by its utilization in multiple studies.^16^, ^17^, ^18^ Furthermore, the ChIA demonstrates cross‐cultural validity in Western (USA)^15^ and Mexican populations (ChIA‐M).^19^ However, its applicability in China's collectivist context—emphasizing group harmony over individual emotional expression^20^, ^21^—remains unverified. Cultural norms discouraging self‐centered emotional displays^22^ may promote anger concealment^23^ and internalization,^24^, ^25^ ^,^ potentially compromising ChIA's item relevance and measurement accuracy in Chinese adolescents. Therefore, it is necessary to examine whether the items of the ChIA scale are suitable for measuring anger in Chinese children and adolescents, and whether the scale effectively captures their experiences with anger.

Developmental variations in anger expression necessitate age‐ and sex‐sensitive assessment. Divergent age‐related patterns emerge, with studies reporting stable self‐reported anger across adolescence^26^ yet increased suppression in older adolescents^27^ and elevated anger among high schoolers.^28^ Sex‐specific expression patterns persist, with boys demonstrating more frequent and intense externalizing aggression versus girls’ internalizing tendencies.^23^, ^29^ These developmental divergences necessitate examining ChIA's measurement invariance across age and sex groups. Furthermore, emerging evidence links elevated anger to depressive trajectories,^30^ yet the ChIA's diagnostic precision for detecting anger‐depression comorbidity in clinical adolescent populations remains unestablished. This gap underscores the critical need to validate the scale's sensitivity and discriminative power in adolescents with psychopathology.

This study aimed to: (1) explore the factor structure of the Chinese version of ChIA (ChIA‐C) in adolescents (11–18 years); (2) establish its psychometric adequacy (reliability, validity); (3) test measurement invariance across age and sex subgroups; and (4) evaluate clinical sensitivity between depressed and healthy adolescents. We hypothesized that the ChIA‐C would retain the original factor structure with potential cross‐cultural adaptations, demonstrate robust psychometric properties, show measurement invariance, and effectively differentiate clinical status in depression screening contexts.

## METHODS

### Ethical approval

This study was approved by the Ethics Committee of the East China Normal University (approval number: HR2‐0066‐2022). All participants provided signed informed consent, and their parents completed a parental notification form.

### Participants

The study recruited 1790 adolescents (57.9% female; mean age of 14.56 ± 1.71 years, range: 11–18 years) from five Chinese provinces (Guangxi, Guangdong, Anhui, Chongqing, and Sichuan). A longitudinal subsample (*n* = 901; 59.2% female; mean age of 13.95 ± 1.66 years) completed 9‐month retesting. Clinical validation involved a case‐control cohort: 50 healthy controls (60% female; mean age of 15.40 ± 0.08 years) and 50 DSM‐5 diagnosed depressed adolescents (76% female; mean age of 14.80 ± 0.25 years) from two tertiary psychiatric hospitals, a district‐level mental health institution.

### Measures

#### Children's Inventory of Anger

The ChIA is a 39‐item self‐report measurement developed by Nelson & Finch^15^ to assess the anger of children and adolescents, using a 4‐point pictorial Likert scale (“I don't care” to “I can't stand that”). The ChIA scale yields a total score and four subscale scores, including FRUST, PHYS, PEER, and AUTH. It demonstrates excellent reliability (total Cronbach's α = 0.95, subscale Cronbach's α = 0.85–0.87).^15^

The Chinese adaptation followed rigorous translation protocols: forward translation by bilingual psychology postgraduate students, back‐translation by an experienced Hong Kong psychologist, and final approval by two psychology professors.

A five‐member expert committee (three psychology professors and two psychology postgraduate students) reviewed the translation. To optimize linguistic appropriateness, modifications were implemented. For instance, culture‐specific metaphors (e.g., “play ‘Keep Away’ with it” in Item 35) were converted into explicit behavioral descriptions in Chinese contexts. Item 35 was thus rephrased as “Two older children deliberately snatch your basketball and prevent you from retrieving it”, while the soda in Item 15 was replaced by the culture‐neutral term “beverage”. Additionally, the original pictorial response scale was simplified to a 4‐point textual format (1 = don't care; 2 = bothers me; 3 = really mad; 4 = furious) for a quicker and better understanding of Chinese adolescents. The original English version and its translated counterpart can be found in Table S1. The four pictorial response options are presented in Figure S1.

#### Anger Expression Scale for Children

The Anger Expression Scale for Children (AESC)^18^ is a self‐report instrument developed for children and adolescents. It comprises 26 items and employs a 4‐point Likert response format ranging from 1 (almost never) to 4 (almost always). The scale yields four subscale scores: Trait Anger, Anger Expression/Out, Anger In/Hostility, and Anger Control/Suppression. In this study, the Chinese version of the AESC scale demonstrated a Cronbach's α of 0.87 and is included for testing convergent validity.

#### Motivation and Pleasure Scale‐Self Report

The Motivation and Pleasure Scale‐Self Report (MAP‐SR) is a 15‐item self‐report tool, created by Llerena et al.,^31^ designed to evaluate amotivation and anhedonia commonly observed in negative symptoms. Each item is assessed on a 5‐point Likert scale, where higher scores indicate more pronounced dysfunction in motivation and pleasure. This study utilizes the Chinese version of the MAP‐SR^32^ to examine discriminant validity. Cronbach's α for the scale was 0.92 in this study.

#### Children's Depression Inventory

The Children's Depression Inventory (CDI) is a self‐report measure used to assess depressive symptoms in children and adolescents. It consists of 27 items and was adapted by Kovacs^33^ from Beck's Depression Inventory (BDI) for adults. The CDI scale includes five dimensions: anhedonia, negative mood, negative self‐esteem, ineffectiveness, and interpersonal problems. Respondents rate each item on a 3‐point Likert scale ranging from 0 to 2. It has been revised and validated in Chinese children^34^ and was included to establish discriminant validity. Cronbach's α for the Chinese version of the CDI was 0.91 in this study.

### Procedure

Participants completed questionnaires, and a follow‐up survey was conducted 9 months later to assess test‐retest stability. A token reward of 5 yuan was provided to participants for completing the initial questionnaire, with an additional incentive for participating in the follow‐up survey. Participation was voluntary, and participants had the right to decline or withdraw at any time. All collected data were handled confidentially and used solely for academic research purposes.

### Data analyses

Out of the initial sample of 1790 participants, 1000 completed the AESC scale, and 1724 completed the MAP‐SR scale. Additionally, five participants (0.3%) had missing values on the CDI scale, for which mean imputation was employed to address the missing data. For the normality testing (Table S2), the scores of each ChIA‐C item were approximately normally distributed with the absolute values of the kurtosis and skewness within 1.50.^35^

We employed both Exploratory Factor Analysis (EFA) and Confirmatory Factor Analysis (CFA) to evaluate the factor structure of the ChIA‐C. The total sample (*n* = 1790) was randomly split into two segments. The first sample (*n* = 790) underwent EFA using Principal Axis Factoring Analysis with Varimax rotation in SPSS 21.0. Items demonstrating poor psychometric properties (low communalities < 0.30, low factor loadings < 0.40, or multiple cross‐loadings) were removed during the EFA process.^19^ The second sample (*n* = 1000) was utilized for CFA, conducted in Mplus 7.4,^36^ to validate the factor structure. Acceptable model fits were defined as follows: Comparative Fit Index (CFI) ≥ 0.90, Tucker‐Lewis Index (TLI) ≥ 0.90, and Root Mean Square Error of Approximation (RMSEA) ≤ 0.10.^37^, ^38^, ^39^

Internal consistency reliability was assessed with a sample of 1790 participants to evaluate the correlation between items within a scale, using Cronbach's α. Test‐retest reliability was examined with 901 participants using Pearson's correlation coefficient and intraclass correlation coefficient (ICC) between wave one and wave two measurements, with *P* < 0.05 used as a significant level. Convergent validity, indicating a significant correlation between two theoretically related measures, was assessed by computing Pearson's correlation coefficients between the ChIA‐C and the AESC. Discriminant validity was determined by examining the relationship between the scores of the ChIA‐C and the Motivation and Pleasure Scale‐Self Report (MAP‐SR), with a negligible correlation supporting discriminant validity. Concurrent validity was established by correlating ChIA‐C scores with scores from the CDI in the initial wave. Predictive validity was demonstrated by correlating ChIA‐C scores from wave one with CDI scores from wave two.

Participants were stratified into developmental subgroups: early adolescence (11–14 years) and middle adolescence (15–18 years) based on neurodevelopmental benchmarks.^6^, ^40^, ^41^, ^42^ To investigate whether measurement properties are consistent across different sex and age groups, measurement invariance across sex and age was conducted in Mplus 7.4.^39^ Configural, metric, scalar, and strict invariance tests were conducted, with criterion values including a ΔCFI ≤ 0.01 and ΔRMSEA ≤ 0.015.^43^, ^44^, ^45^, ^46^ In cases where invariance was not supported, model modification indices were used to identify sources of partial measurement invariance.^47^ Additionally, Independent *t‐*tests were conducted for age and sex to examine score differences on the ChIA‐C scale between groups, offering insights into the scale's reliability across sex and age. Moreover, additional *t*‐tests were performed to examine the ability of the ChIA‐C scale to differentiate between healthy and clinically depressed adolescent groups.

## RESULTS

### Factor structure of the ChIA‐C

An EFA was conducted with a sample of 790 participants. The suitability of the data for EFA was confirmed by the Kaiser‐Meyer‐Olkin index (0.94) and Bartlett's sphericity test ($\chi_{300}^{2}$= 10945.13, *df* = 741, *P* < 0.001). Factor extraction and rotation revealed psychometric limitations in 12 items that necessitated removal. Items 8, 13, and 39 exhibited low communalities (<0.30), while items 7, 17, 22, and 29 showed both low communalities (<0.30) and low factor loadings (<0.40). Additionally, multiple cross‐loadings were observed for items 1, 2, 16, 18, and 21. Consequently, these 12 items were excluded from subsequent analyses (Table S2). With the remaining 27 items, the ChIA‐C revealed a four‐factor structure based on eigenvalues > 1 and the Scree plot. This structure accounted for a total variance of 69.98% (Table S3). The subscales closely mirrored those of the original ChIA, encompassing PHYS, FRUST, AUTH, and PEER.

**FIGURE 1:**
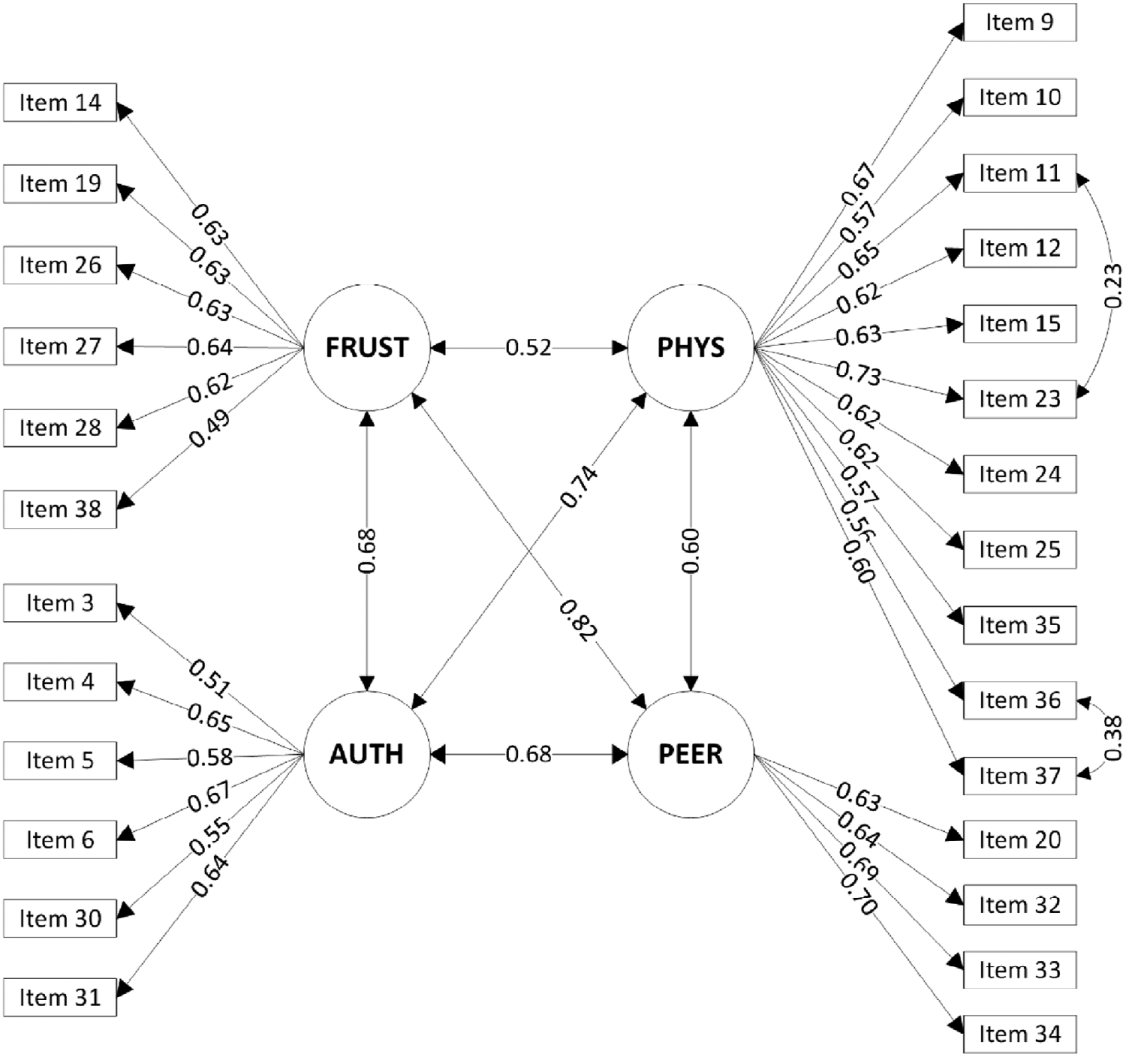
Confirmatory factor analysis of the Chinese version of the Children's Inventory of Anger. FRUST, frustration; PHYS, physical aggression; PEER, peer relationships; AUTH, authority relations.

Moreover, a CFA was conducted using a separate sample of 1000 participants. The revised four‐factor structure exhibited satisfactory model fit ($\chi^{2}$= 1053.76, *df* = 315, $\chi^{2}/df$= 3.35, CFI = 0.92, TLI = 0.91, and RMSEA = 0.05) (Figure 1 and Table 1), in contrast to the inadequate fit indices observed for the original 39‐item scale's four‐factor model ($\chi^{2}$ = 3481.61, *df* = 696, $\chi^{2}/df$ = 5.00, CFI = 0.79, TLI = 0.76, and RMSEA = 0.06). Additionally, correlations between each item and the total score were computed (Table S3). The results indicated that the correlation coefficient between each of the 27 items and the total score of the scale ranged from 0.49 to 0.72 (*P* < 0.010), indicating good construct validity.

**TABLE 1:** Confirmatory factor analysis model fitting index

*n* = 1000	χ^2^	df	χ^2^/df	CFI	TLI	RESEA	SRMR
**Four‐factor (39 items** ^†^)	3481.61	696	5.00	0.79	0.78	0.06	0.07
**Four‐factor (27 items** ^‡^)	1082.74	316	3.43	0.92	0.91	0.05	0.05

*χ^2^*, chi‐square test of model fit; *df*, degree of freedom; CFI, comparative fit index; TLI, Tucker‐Lewis index; RMSEA, root mean square error of approximation; SRMR, standardized root means square residual.

^†^ 39‐item original Children's Inventory of Anger (ChIA).

^‡^ 27‐item Chinese version of the ChIA with 12 items deleted from the original ChIA.

### Reliability analysis

Cronbach's α for the ChIA‐C total score was 0.92, indicating excellent internal consistency. For the four subscales—FRUST, PHYS, PEER, and AUTH—Cronbach's α were 0.76, 0.88, 0.77, and 0.77, respectively, demonstrating satisfactory internal reliability (Table 2). Additionally, Pearson correlation coefficients and intraclass correlation coefficients between wave one and wave two, across the 9‐month interval, ranged from 0.38 to 0.50 (Table 2), indicating a moderate level of consistency and stability in measuring the ChIA‐C between the two time points of 9 months.

**TABLE 2:** The internal consistency, reliability, and test‐retest reliability of ChIA‐C and ChIA

	Internal consistency reliability (*n* = 1790)		Test‐retest reliability (*n* = 901)		
	**Cronbach's α**		**PCC**		**ICC**
Score	ChIA‐C	ChIA ^†^	ChIA‐C	ChIA ^‡^	ChIA‐C
**FRUST**	0.76	0.85	0.40^***^	0.73	0.40^***^
**PHYS**	0.88	0.86	0.48^***^	0.69	0.48^***^
**PEER**	0.77	0.86	0.38^***^	0.65	0.38^***^
**AUTH**	0.77	0.85	0.48^***^	0.75	0.48^***^
**Total score**	0.92	0.95	0.50^***^	0.75	0.50^***^

FRUST, frustration; PHYS, physical aggression; PEER, peer relationships; AUTH, authority relations; ChIA, Children's Inventory of Anger; ChIA‐C, Chinese version of the ChIA; PCC, Pearson correlation coefficients; ICC, intraclass correlation coefficients.

^†^ The original sample of the ChIA scale consists of 1604 participants aged 6–16 years.

^‡^ The original sample of the ChIA scale consists of 87 participants aged 6–11 years with a 1‐week interval.^15^

*** *P* < 0.001.

### Validity analysis

#### Convergent validity and discriminant validity

**FIGURE 2:**
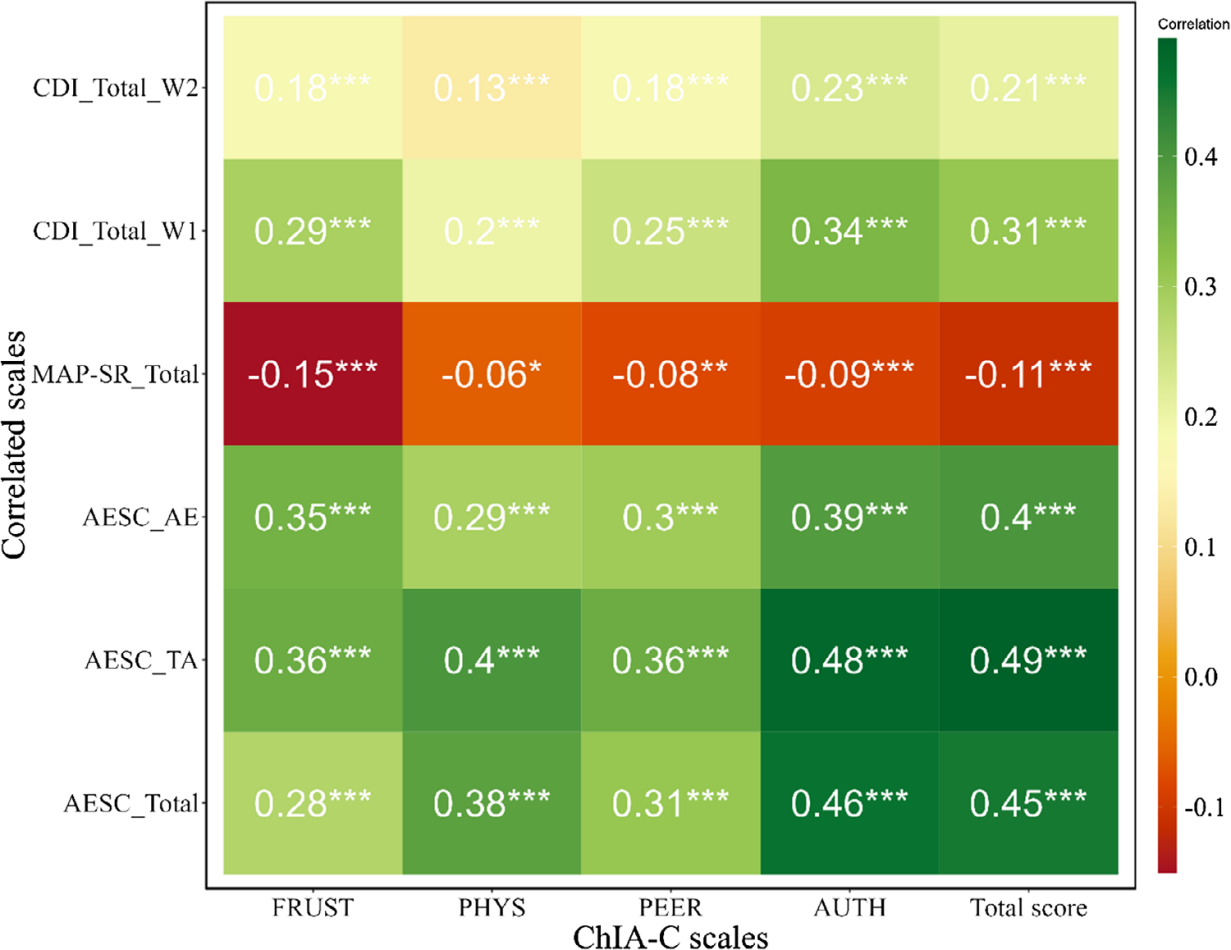
Correlations between the ChIA‐C and convergent (AESC, *n* = 1000), discriminant measures (MAP‐SR, *n* = 1724), and concurrent and predictive measures (CDI, *n* = 1790). ChIA‐C, Chinese version of the Children's Inventory of Anger; FRUST, frustration subscale; PHYS, physical aggression subscale; PEER, peers relationships subscale; AUTH, authority relations subscale; Total score, total score of the ChIA‐C; AESC, Anger Expression Scale for Children; TA, trait anger subscale; AE, anger expression subscale; MAP‐SR, Motivation and Pleasure Scale‐Self Report; CDI, Children's Depression Inventory. W1: data collected in the first wave. W2: data collected in the second wave (9‐month interval). ^*^ *P* < 0.05, ^**^ *P* < 0.01, ^***^ *P* < 0.001.

To evaluate the convergent validity of the ChIA‐C, correlations were computed between ChIA‐C scores and AESC total score, as well as its two subscale scores (trait anger and anger expression) in a sample of 1000 participants. The results indicated significant positive correlations (*r* = 0.28–0.49, *P* < 0.001, Figure 2) between the total scores of the two scales, suggesting a robust association between the ChIA‐C and AESC scales in assessing anger feelings, indicative of good convergent validity.

Discriminant validity was assessed by examining correlations between ChIA‐C scores and the MAP‐SR total score. Negligible negative correlations were observed (Figure 2, *r* = −0.15, −0.09, and −0.11, respectively, *P* < 0.001; *r* = −0.06, *P* = 0.011; *r* = −0.08, *P* = 0.001), implying conceptual distinctiveness between the ChIA‐C and MAP‐SR. The MAP‐SR predominantly focuses on measuring self‐reported pleasant experiences, contrasting with the ChIA‐C's focus on anger, thus demonstrating adequate discriminant validity for the ChIA‐C.

#### Concurrent validity and predictive validity

The CDI was employed to evaluate concurrent and predictive validity. The ChIA‐C scores demonstrated significant moderate correlations with the CDI total score at wave one (*r* = 0.20–0.34, *P* < 0.001, Figure 2), indicating acceptable concurrent validity. Furthermore, the correlation between the ChIA‐C scale at wave one and CDI at wave two remained significant, with small to moderate effects (*r* = 0.13–0.23, *P* < 0.001). These findings suggest acceptable concurrent validity and moderate predictive validity.

#### Measurement invariance

Measurement invariance across age was confirmed, exhibiting satisfactory model fit indices (Table 3, ΔCFI ≤ 0.01, ΔRMSEA ≤ 0.015) in all models. This suggests that the structure, factor loadings, and item intercepts of the ChIA‐C scale remain consistent across both early adolescence (ages 11–14 years) and middle adolescence (ages 15–18 years).

**TABLE 3:** Measurement invariance of the Chinese version of the Children's Inventory of Anger across sex and age

Model	χ^2^	df	RMSEA	CFI	TLI	SRMR	ΔRMSEA (M0‐M1)	ΔCFI (M0‐M1)	ΔTLI (M0‐M1)	ΔSRMR (M0‐M1)
**Across age (*n*_aged 11–14_ = 692, *n*_aged 15–18_ = 1098)**										
M0 (Configural)	1838.23	630	0.046	0.911	0.901	0.048				
M1 (Metric/Weak)	1898.45	653	0.046	0.909	0.902	0.051	0.000	0.002	−0.001	−0.003
M2 (Scalar/Strong)	1982.67	676	0.046	0.904	0.900	0.052	0.000	0.005	0.002	−0.001
M3 (Strict)	2034.86	703	0.046	0.902	0.902	0.053	0.000	0.002	−0.002	−0.001
**Across sex (*n*_female_ = 1037, *n*_male_ = 753)**										
M0 (Configural)	1839.02	630	0.046	0.91	0.899	0.049				
M1 (Metric/Weak)	1873.71	653	0.046	0.909	0.902	0.050	0.000	0.001	−0.003	−0.001
M2 (Scalar/Strong)	2154.90	676	0.049	0.889	0.885	0.054	−0.003	0.02	0.017	−0.004
M3 (Strict)	2268.93	703	0.050	0.883	0.883	0.055	−0.001	0.006	0.002	−0.001
M2^†^ (Scalar/Strong)	2004.03	668	0.047	0.900	0.895	0.051	−0.001	0.009	0.007	−0.001

*χ^2^*, chi‐square test of model fit; *df*, degree of freedom; CFI, comparative fit index; TLI, Tucker‐Lewis index; RMSEA, root mean square error of approximation; SRMR, standardized root means square residual; M0 (configural), configural invariance test; M1 (Metric/Weak), metric invariance test; M2 (Scalar/Strong), scalar invariance test; M3 (Strict), strict invariance test.

^†^ Scalar invariance model with the intercepts of four items (items 9, 11, 23, and 25) released.

Concerning the factor of sex, both configural invariance and metric invariance were confirmed, but some variation was observed at the scalar level. Therefore, partial invariance was implemented by releasing constraints to address non‐invariance across sex groups.^39^, ^47^, ^48^, ^49^ Significant differences were observed in the intercept (τ) of item 9 (male: τ = 2.86, *P* < 0.001, female: τ = 3.51, *P* < 0.001), item 11 (male: τ = 3.52, *P* < 0.001, female: τ = 4.32, *P* < 0.001), item 23 (male: τ = 2.99, *P* < 0.001, female: τ = 3.70, *P* < 0.001), and item 25 (male: τ = 2.57, *P* < 0.001, female: τ = 3.04, *P* < 0.001). With these 4 item intercepts freed, the model fit indices were satisfactory (ΔCFI ≤ 0.01, ΔRMSEA ≤ 0.015), indicating acceptable partial measurement invariance.

Moreover, the *t*‐test outcomes (Tables S4 and S5) suggest that, in general, there were no significant score differences between early and middle adolescents. However, for sex differences, girls scored significantly higher than boys on all items and subscales (*P* < 0.001).

### Sensitivity analysis of clinical application

**FIGURE 3:**
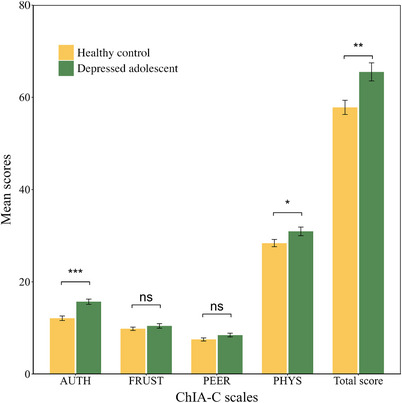
The *t*‐tests of the ChIA‐C scores between healthy and clinically depressed adolescent groups. ChIA‐C, Chinese version of the Children's Inventory of Anger; FRUST, frustration subscale; PHYS, physical aggression subscale; PEER, peer relationships subscale; AUTH, authority relations subscale; Total score, total score of the ChIA‐C; ns, not significant. ^*^ *P* < 0.05, ^**^ *P* < 0.01, ^***^ *P* < 0.001.

The *t*‐test results indicated significant differences between the depressed group (mean = 65.52) and the healthy group (mean = 57.82) on the ChIA‐C total score, with a statistically significant effect (*t* (98) = 3.09, *P* = 0.003, Cohen's *d* = 0.62, Figure 3). Additionally, significant differences were observed between the two groups on the AUTH (*t* (98) = 4.76, *P* < 0.001, Cohen's *d* = 0.95) and PHYS subscales (*t* (98) = 2.08, *P* = 0.040, Cohen's *d* = 0.42).

## DISCUSSION

This study validates the psychometrics of the 27‐item ChIA‐C. The results of both EFA and CFA provided support for a four‐factor structure: FRUST, PHYS, PEER, and AUTH. The ChIA‐C exhibited strong internal consistency and test‐retest reliability. Additionally, it demonstrated satisfactory convergent and divergent validity, as well as concurrent and predictive validity. Measurement invariance across age groups was established, while partial measurement invariance was observed for sex. Moreover, the ChIA‐C scale exhibited sensitivity in distinguishing between healthy and depressed groups in clinical applications. These results collectively suggest that the ChIA‐C is a suitable instrument for assessing anger in Chinese children and adolescents.

### Factor structure of the ChIA‐C

Exploratory and confirmatory factor analyses validated the ChIA‐C's four‐factor structure (i.e., FRUST, PHYS, PEER, and AUTH), albeit with 12 unsuitable items removed. Modifications mirrored adaptations in ChIA‐M, driven by psychometric and conceptual considerations.

Items 1, 2, 16, and 18 were removed due to cross‐loadings on both the AUTH and FRUST factors, indicating ambiguous construct measurement and insufficient discriminant validity. Within Chinese cultural contexts, compliance with parental and academic authority is normatively sanctioned.^50^ Consequently, while Chinese adolescents typically accept interventions in scenarios like item 1 (Your mother calls you to dinner in the middle of your favorite TV show) and Item 16 (A teacher gives you a lot of homework on the weekend), these situations simultaneously elicit frustration from perceived deprivation of leisure autonomy.^51^ Items 2 (Your bike has a flat tire) and 18 (Someone turns the TV to another channel when you are watching a show), though framed as personal property violations in Western contexts, reflect distinct cultural characteristics in China where household resources like children's transportation and television are often purchased and governed by parents,^50^ Item 2 may evoke anxiety about authority criticism for perceived irresponsibility, while item 18 primarily signifies entertainment disruption. Lastly, item 21 (Somebody says “I told you so” after something goes wrong) was excluded due to cross‐loadings on AUTH, PHYS, and PEER, eliciting predominantly shame responses rather than prototypical anger in Chinese youth.^52^ This ambiguity in anger attribution compromised the item's discriminant validity, necessitating its removal.

The remaining seven items (7, 8, 13, 17, 22, 29, and 39) were deleted due to low communalities or factor loadings. Items representing autonomy restrictions (17: “Someone says that you are not old enough to do something”; 22: “Your mom or dad slaps you”; 29: “Your mom says she does not want you to play with one of your friends”; 39: “You have to go to bed at 9:30 and your friends get to stay up until 10:30 or 11:00”) align with western individualistic conceptions of rights violations,^53^ yet Chinese children's acceptance of familial authority^54^ and interpretation of parental controls as protective^55^ diminished their measurement efficacy. Similarly, Item 8 (In a game, someone on the other side tries to cheat) provoked weaker reactions in contexts prioritizing relational harmony,^56^ while Items 7 (The teacher's pet gets to do all the fun jobs in class) and 13 (Somebody calls you a “chicken”) predominantly elicited shame rather than anger in collectivist settings,^57^ compromising their construct representation.

### Validity, reliability, and sensitivity analysis of the ChIA‐C

The ChIA‐C scale demonstrated convergent validity through significant positive correlations with the “anger expression” and “trait anger” subscales of the AESC scale, paralleling the ChIA‐M's associations with trait anger measures.^19^ Discriminant validity was established by significantly negligible negative correlations with the MAP‐SR scale, confirming anger‐specific measurement. This finding parallels the ChIA‐M's negative association with positive emotion measures^19^ and the original ChIA's discriminative capacity in incarcerated versus normative populations.^15^ Moreover, the ChIA‐C scale demonstrated moderate concurrent validity and weak predictive validity through baseline and 9‐month follow‐up correlations with CDI scores, consistent with the established finding that anger is theoretically linked to depression.^15^, ^58^

Regarding reliability, the ChIA‐C demonstrated excellent internal consistency; similar results can be seen in the original versions of the ChIA^15^ and ChIA‐M.^19^ The 9‐month test‐retest reliability was demonstrated by satisfactory Pearson and intraclass correlation coefficients. Moreover, the original ChIA scale assessed test‐retest reliability with a 1‐week interval. Both sets of findings collectively evidence robust measurement consistency across extended developmental periods.

Critically, while initial validation focused on healthy adolescents, a clinical extension study employing a case‐control design revealed significantly higher ChIA‐C scores in depressed adolescents than in healthy controls. This clinically meaningful divergence confirms the scale's diagnostic discriminative capacity in pediatric mood disorder assessments.

### Measurement invariance of the ChIA‐C

The ChIA‐C demonstrated full measurement invariance across early (11–14 years) and middle adolescence (15–18 years), confirming configural, metric, and scalar invariance. This psychometric rigor ensures equivalent construct interpretation across developmental stages,^59^ addressing a critical limitation of the original ChIA, which lacked formal invariance testing. To our knowledge, this constitutes the first cross‐validated evidence of age‐related measurement invariance in the ChIA scale, enabling reliable detection of latent mean differences.

In terms of sex‐based measurement invariance, the ChIA‐C demonstrated metric invariance across sexes, confirming equivalent factor structures between males and females. However, scalar invariance was not achieved even after imposing equal factor loadings across groups, primarily due to intercept differences.^60^ Partial invariance required freeing intercepts of four items (items 9, 11, 23, and 25 from the PHYS factor) with the largest χ^2^ residuals, which exhibited higher intercepts for females, revealing elevated female intercepts despite comparable PHYS levels. Chinese girls may underreport overt aggression due to gendered socialization emphasizing restraint, while elevated intercepts could reflect internalized anger manifesting as somatic complaints.^61^, ^62^ Additionally, Items describing direct PHYS (e.g., item 25: “Someone punches you”) may evoke differential interpretations, with females perceiving indirect or relational hostility as more salient, inflating scores despite comparable behavioral frequencies.^23^ Therefore, Researchers should verify invariance in their data when investigating sex differences,^59^ particularly for cross‐cultural aggression studies using these items.

### Limitations and future research

While the ChIA‐C has demonstrated good reliability and validity among Chinese children and adolescents, it is important to acknowledge certain limitations warranting further investigation. Initially, the present study utilized a relatively small clinical sample of 50 adolescents with depression. While providing initial insights, this sample size may limit the generalizability of findings and was insufficient for conducting robust factor analysis to definitively evaluate the measure's factor structure within this population. Future research should prioritize replicating these findings in larger, independent clinical samples of adolescents with depression to confirm the factor structure and enhance generalizability. Furthermore, the test‐retest interval of 9 months, though necessitated by coronavirus disease 2019‐related protocol deviations, exceeds the typical windows recommended for establishing psychometric stability in psychological constructs, particularly in developmental or pediatric contexts. Future studies should implement shorter intervals (e.g., 1–3 months) per pediatric psychometric norms. Another limitation is the absence of cognitive interviews with children during translation. While the expert committee review partially assessed item comprehension, this approach could not verify developmental nuances in children's interpretations. Future work should prioritize direct cognitive testing with stratified age groups. Finally, while the ChIA‐C scale has shown significant clinical sensitivity, its capacity to track treatment effects requires validation across China's diverse subpopulations, particularly in culturally distinct regions.

## Conclusion

The ChIA‐C maintains a comparable 4‐factor structure to the original version, albeit with some items deleted or modified. Our findings reveal good reliability and acceptable validity, demonstrating measurement invariance across different age groups. Additionally, the scale shows sensitivity in distinguishing between healthy and depressed groups in clinical applications. Taken together, these results indicate that the ChIA‐C is well‐suited for use with Chinese children and adolescents. In practical applications, it can be instrumental in experimental studies assessing subjective anger emotions and can contribute to evaluating the effectiveness of psychotherapeutic and educational interventions for anger management.

## CONFLICT OF INTEREST

The authors declare no conflict of interest.

## Supporting information



Supporting Information
