# Supplementary material for: Evaluating anger in Chinese adolescents: Psychometric validation and clinical sensitivity of the Chinese version of the Children's Inventory of Anger
Source: Pediatr Investig. 2025 Oct 11;10(2):147–58. doi: 10.1002/ped4.70024 (PMC13109863; doi:10.1002/ped4.70024)
Supplement: Supplementary file 1 — Supporting Information [file PED4-10-147-s001.pdf]

Supplementary Material for

Evaluating anger in Chinese adolescents: Psychometric validation and clinical sensitivity of the Chinese version of the Children's Inventory of Anger

Xinyue Zhang, Liping Ma, Chi-son Kuan, Xinli Chi, Yuan Cao, Suhong Wang, Chao Yan, W. Michael Nelson III

Table S1 The children's inventory of anger (English version and Chinese version)

Item[a]-ChIA	Item[b]-ChIA-C	English original version	Chinese item
1	Excluded	Your mother calls you to dinner in the middle of your favorite TV show	当你正在看你最喜欢的电视节目时,你妈妈叫你去吃晚饭
2	Excluded	Your bike has a flat tire	你的自行车爆胎了
3	1	Your brother or sister or friend ignores you	你的兄弟、姐妹或者朋友无视你
4	2	You clean up your room and want to go out to play, your mom says you have to clean some more	你打扫完房间准备出去玩,你妈妈表示你必须再打扫点别的
5	3	You have to do a job that your brother or sister was supposed to do	你不得不做一些你兄弟或者姐妹应该做的工作
6	4	You want to go somewhere with a friend, Your dad says "No" for no reason	你想和朋友去某个地方,你爸爸毫无理由的拒绝了
7	Excluded	The teacher's pet gets to do all the fun jobs in class	你成为课堂上大家的笑点
8	Excluded	In a game, someone on the other side tries to cheat	游戏中，对方有人试图作弊
9	5	Someone bumps your desk on purpose and you mess up your work	某人故意撞翻你的桌子，让你把工作搞砸了
10	6	You are playing a game and someone on the other side tries to punch you	你正在玩游戏，旁边某人试图打你
11	7	Someone spits at you.	有人向你吐口水
12	8	You get a piece of pie at lunch and someone knocks it out of your hand	你在午餐时得到一块馅饼,然后有人把它从你手里打掉了
13	Excluded	Somebody calls you a "chicken"	有人叫你"胆小鬼"
14	9	You want to show someone a new trick on your bike and you can't do it	你向他人展示你新学的自行车技巧，但你失败了
15	10	You put all your change in the soda machine, It takes your money, but you don't get a drink	你把所有零钱都放进饮料自动贩卖机里,它收了你的钱但没有饮料掉出来

Item[a]-ChIA	Item[b]-ChIA-C	English original version	Chinese item
16	Excluded	A teacher gives you a lot of homework on the weekend	老师在周末给你布置了大量作业
17	Excluded	Someone says that you are not old enough to do something	某人说你还太小，不能做某事
18	Excluded	Someone turns the TV to another channel when you are watching a show	某人在你看电视时换台
19	11	Your brother or sister says “No” when you ask for something	你的兄弟或者姐妹在你向她们请求时说“不”
20	12	Your friends are playing a game and they won’t let you play	你的朋友们在玩游戏并且不让你参加
21	Excluded	Somebody says “I told you so” after something goes wrong	当事情搞砸时，有人对你说“我早就告诉过你了”
22	Excluded	Your mom or dad slaps you	你爸爸或者妈妈拍打你
23	13	Someone puts gum on your seat on the bus and you sit on it	有人把口香糖放在你的座位上，你坐上去了
24	14	You bump into a stranger on the bus. He says he will beat you up if you get near him again	你在公交车上撞到了一个陌生人，他说如果你再靠近他，他就揍你
25	15	Someone cuts in front of you in line	有人在你前面插队
26	16	You brought your favorite candy bar in your lunch. When you go to eat it, it’s melted	你在午餐带了最喜欢的棒棒糖，但当你准备吃它时，它已经化了
27	17	Your mom won’t buy your favorite cereal	你的妈妈不给你买你最喜欢的麦片
28	18	On your bike, you come to a steep hill. You have to get off the bike and walk it all the way up the hill	你骑车时遇到一个非常陡峭的山坡，你不得不下车推行上去
29	Excluded	Your mom says she does not want you to play with one of your friends	你的妈妈说她不希望你和你的某个朋友一起玩
30	19	Your dad yells at you in front of other people	你父亲当着别人的面朝你喊叫
31	20	You don’t have any homework, but your mom makes you study anyway	你没有作业要做但你妈妈强迫你无论如何要学习
32	21	You do something special for a friend and he or she won’t do anything for you	你为朋友做了很特别的事，而对方却什么都没为你做

Item[a]-ChIA	Item[b]-ChIA-C	English original version	Chinese item
33	22	Your friends pick you last to be on a team	你的朋友在组队时最后一个才选你
34	23	Your friend gets what he or she wants for Christmas, but you don’t	你的朋友得到了想要的圣诞礼物但你没有
35	24	Two bigger kids come and take your basketball and play “Keep Away” with it	两个比你大的孩子抢走了你的篮球
36	25	Somebody punches you	有人打你
37	26	Someone tries to trip you on purpose	有人试图故意绊倒你
38	27	You have to do your homework and your brother or sister gets to watch TV	你必须写作业的时候你的兄弟或者姐妹却开始看电视
39	Excluded	You have to go to bed at 9:30 and your friends get to stay up until 10:30 or 11:00	你必须在九点半上床，但你的朋友可以等到十点半或者十一点

Note. [a]Order number of the original 39-item ChIA. [b]New order number of the 27-item ChIA of the Chinese version.

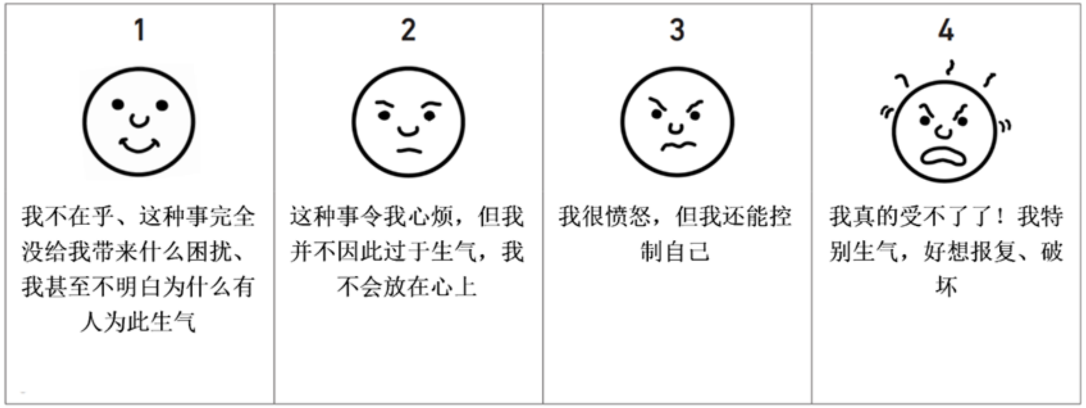

Figure S1 The four ChIA-C response choices and their associated pictures.
1 = don’t care; 2 = bothers me; 3 = really mad; 4 = furious.

Table S2 Items deleted in the Chinese version of the ChIA

Reasons for deleted	Items deleted		Communalities	Factor loadings
Low communalities	8	In a game, someone on the other side tries to cheat	0.27	−0.47
	13	Somebody calls you a “chicken”	0.23	0.40
	39	You have to go to bed at 9:30 and your friends get to stay up until 10:30 or 11:00	0.27	0.39
Low communalities and low factor loadings	17	Someone says that you are not old enough to do something	0.26	0.21
	7	The teacher’s pet gets to do all the fun jobs in class	0.19	0.22
	22	Your mom or dad slaps you	0.23	<0.20
	29	Your mom says she does not want you to play with one of your friends	0.21	0.23
cross loadings	1	Your mother calls you to dinner in the middle of your favorite TV show	0.30	0.41, 0.30
	2	Your bike has a flat tire	0.27	0.28, 0.26
	16	A teacher gives you a lot of homework on the weekend	0.23	0.22, −0.20, 0.21
	18	Someone turns the TV to another channel when you are watching a show	0.35	0.27, 0.26
	21	Somebody says “I told you so” after something goes wrong.	0.35	0.21, −0.21, −0.20

Table S3 Results of the EFA, CFA and item-total correlations of the 27-item ChIA

Item	*M*	*SD*	*Skewness*	*Kurtosis*	Factor				CFA	Item-total correlation
					PHYS-11	FRUST-6	AUTH-6	PEER-4	λ	
**9**	2.84	0.87	−0.47	−0.38	0.51				0.67	0.62**
**10**	2.35	0.94	0.12	−0.90	0.46				0.59	0.57**
**11**	3.23	0.81	−0.97	0.59	0.60				0.65	0.60**
**12**	2.49	0.92	−0.05	−0.83	0.54				0.62	0.60**
**15**	2.5	0.84	0.17	−0.57	0.50				0.63	0.61**
**23**	2.97	0.87	−0.58	−0.30	0.60				0.74	0.65**
**24**	2.4	0.92	0.18	−0.79	0.45		−0.35		0.62	0.61**
**25**	2.54	0.88	−0.17	−0.67	0.63				0.62	0.59**
**35**	2.33	0.97	0.05	−1.03	0.50				0.56	0.56**
**36**	2.78	0.90	−0.32	−0.68	0.76				0.56	0.56**
**37**	2.69	0.92	−0.31	−0.70	0.72				0.60	0.59**
**14**	1.56	0.64	1.01	1.27		0.45			0.63	0.47**
**19**	1.72	0.71	0.77	0.43		0.35			0.63	0.55**
**26**	1.56	0.71	1.26	1.48		0.68			0.63	0.49**
**27**	1.51	0.67	1.24	1.35		0.68			0.64	0.47**
**28**	1.49	0.71	1.42	1.63		0.60			0.62	0.45**
**38**	1.74	0.83	0.86	−0.10		0.38			0.49	0.48**
**3**	2.02	0.76	0.36	−0.26			−0.46		0.51	0.52**
**4**	1.99	0.78	0.35	−0.48			−0.70		0.65	0.56**
**5**	1.92	0.84	0.55	−0.44			−0.50		0.58	0.52**
**6**	2.28	0.81	0.16	−0.47			−0.67		0.67	0.60**

Item	*M*	*SD*	*Skewness*	*Kurtosis*	Factor				CFA	Item-total correlation
					PHYS-11	FRUST-6	AUTH-6	PEER-4	λ	
**30**	2.13	0.92	0.38	−0.75			−0.34		0.55	0.55**
**31**	2.01	0.84	0.56	−0.22			−0.31		0.64	0.57**
**20**	2.04	0.81	0.43	−0.34				−0.35	0.63	0.60**
**32**	1.85	0.82	0.66	−0.25				−0.46	0.64	0.57**
**33**	1.74	0.85	0.91	−0.03				−0.49	0.69	0.59**
**34**	1.61	0.74	1.13	1.03				−0.49	0.70	0.56**
**Percentage of variance explained (%)**					23.09	15.88	19.72	11.28		
**Factorial correlation matrix**					**PHYS**	**FRUST**	**AUTH**	**PEER**		
**PHYS**					1.00					
**FRUST**					0.34	1.00				
**AUTH**					−0.54	−0.44	1.00			
**PEER**					−0.33	−0.37	0.29	1.00		

Note. Item, order number of the original ChIA; M, mean; SD, standard deviation; CFA, confirmatory factor analysis; λ, factor loadings from the CFA; FRUST-11, frustration factor with 11 items; PHYS-6, physical Aggression factor with 6 items; AUTH-6, authority relations factor with 6 items; PEER-4, peers relationships factor with 4 items.

Factor loadings with absolute values less than 0.3 are not presented in the table.

$^{*}P < 0.05$; $^{**}P < 0.01$; $^{***}P < 0.001$.

Table S4 The *t*-test of the ChIA scores across age groups

Items and scales	Early Adolescence (n=692) *M(SD)*	Middle Adolescence (n=1098) *M(SD)*	*t*	*P*	Mean Difference	95% Confidence Interval
CHIA_3	2.01(0.76)	2.03(0.75)	−0.55	0.584	−0.02	−0.09~0.05
CHIA_4	1.93(0.81)	2.04(0.76)	−2.84	0.004	−0.11	−0.19~−0.03
CHIA_5	1.84(0.84)	1.98(0.83)	−3.51	<0.001	−0.14	−0.22~−0.06
CHIA_6	2.19(0.81)	2.33(0.8)	−3.65	<0.001	−0.14	−0.22~−0.07
CHIA_9	2.84(0.91)	2.83(0.85)	0.21	0.829	0.01	−0.08~0.09
CHIA_10	2.36(0.98)	2.34(0.92)	0.44	0.654	0.02	−0.07~0.11
CHIA_11	3.23(0.75)	3.23(0.84)	0.06	0.950	0	−0.07~0.08
CHIA_12	2.52(0.94)	2.47(0.9)	1.24	0.214	0.06	−0.03~0.14
CHIA_14	1.58(0.64)	1.56(0.64)	0.60	0.548	0.02	−0.04~0.08
CHIA_15	2.48(0.86)	2.52(0.82)	−1.02	0.308	−0.04	−0.12~0.04
CHIA_19	1.68(0.69)	1.75(0.72)	−2.03	0.042	−0.07	−0.14~0
CHIA_20	1.97(0.84)	2.08(0.79)	−2.63	0.008	−0.11	−0.18~−0.03
CHIA_23	2.92(0.87)	3.00(0.87)	−1.82	0.069	−0.08	−0.16~0.01
CHIA_24	2.42(0.94)	2.38(0.91)	0.86	0.391	0.04	−0.05~0.13
CHIA_25	2.52(0.91)	2.55(0.85)	−0.73	0.460	−0.03	−0.12~0.05
CHIA_26	1.53(0.71)	1.58(0.71)	−1.36	0.176	−0.05	−0.12~0.02
CHIA_27	1.51(0.69)	1.50(0.66)	0.04	0.970	0	−0.06~0.07
CHIA_28	1.46(0.70)	1.51(0.72)	−1.33	0.184	−0.05	−0.11~0.02
CHIA_30	1.99(0.96)	2.21(0.88)	−5.11	<0.001	−0.23	−0.31~−0.14
CHIA_31	1.97(0.88)	2.04(0.82)	−1.66	0.092	−0.07	−0.15~0.01
CHIA_32	1.85(0.85)	1.86(0.80)	−0.26	0.791	−0.01	−0.09~0.07

Items and scales	Early Adolescence (n=692) *M(SD)*	Middle Adolescence (n=1098) *M(SD)*	***t***	***P***	**Mean Difference**	**95% Confidence Interval**
CHIA_33	1.65(0.84)	1.80(0.86)	−3.50	<0.001	−0.14	−0.23 to −0.06
CHIA_34	1.64(0.77)	1.59(0.71)	1.25	0.204	0.05	−0.03 to 0.12
CHIA_35	2.40(0.98)	2.29(0.96)	2.4	0.016	0.11	0.02 to 0.20
CHIA_36	2.76(0.90)	2.79(0.91)	−0.62	0.536	−0.03	−0.11 to 0.06
CHIA_37	2.75(0.91)	2.65(0.92)	2.27	0.024	0.10	0.01 to 0.19
CHIA_38	1.75(0.81)	1.73(0.85)	0.61	0.539	0.03	−0.05 to 0.10
CHIA_FRUST	9.51(2.91)	9.63(2.90)	−0.84	0.403	−0.12	−0.39 to 0.16
CHIA_PHYS	29.20(6.69)	29.03(6.51)	0.51	0.612	0.16	−0.46 to 0.79
CHIA_PEER	7.11(2.55)	7.32(2.41)	−1.79	0.073	−0.21	−0.45 to 0.02
CHIA_AUTH	11.92(3.43)	12.62(3.29)	−4.37	< 0.001	−0.71	−1.03 to −0.39
CHIA_Total	57.73(12.72)	58.61(12.38)	−1.45	0.148	−0.88	−2.07 to 0.31

Note. M, mean; *SD*, standard deviation; early adolescence, 11–14 years; middle adolescence, 15–18 years; FRUST, frustration subscale; PHYS, physical aggression subscale; PEER, peers relationships subscale; AUTH, authority relations subscale.

Table S5 The *t*-test of the ChIA scores across gender

Items and scales	Male(n=753) *M(SD)*	Female(n=1037) *M(SD)*	*t*	*P*	Mean Difference	95% Confidence Interval
CHIA_3	1.9(0.76)	2.11(0.74)	−5.86	<0.001	−0.21	−0.28 to −0.14
CHIA_4	1.89(0.77)	2.07(0.78)	−4.86	<0.001	−0.18	−0.25 to −0.11
CHIA_5	1.75(0.80)	2.05(0.84)	−7.55	<0.001	−0.30	−0.38 to −0.22
CHIA_6	2.14(0.81)	2.38(0.78)	−6.20	<0.001	−0.24	−0.31 to −0.16
CHIA_9	2.67(0.95)	2.96(0.79)	−6.77	<0.001	−0.29	−0.37 to −0.21
CHIA_10	2.35(0.98)	2.34(0.91)	0.18	0.854	0.01	−0.08 to 0.10
CHIA_11	3.11(0.90)	3.31(0.72)	−5.14	<0.001	−0.20	−0.28 to −0.13
CHIA_12	2.47(0.95)	2.50(0.89)	−0.53	<0.001	−0.02	−0.11 to 0.06
CHIA_14	1.49(0.67)	1.62(0.62)	−4.24	<0.001	−0.13	−0.19 to −0.07
CHIA_15	2.49(0.88)	2.51(0.80)	−0.63	0.522	−0.03	−0.11 to 0.05
CHIA_19	1.69(0.73)	1.74(0.70)	−1.40	0.163	−0.05	−0.11 to 0.02
CHIA_20	1.93(0.82)	2.12(0.80)	−4.92	<0.001	−0.19	−0.27 to −0.11
CHIA_23	2.72(0.94)	3.15(0.77)	−10.44	<0.001	−0.44	−0.52 to −0.35
CHIA_24	2.33(0.97)	2.44(0.89)	−2.46	0.013	−0.11	−0.20 to −0.02
CHIA_25	2.44(0.95)	2.60(0.81)	−3.69	<0.001	−0.16	−0.24 to −0.07
CHIA_26	1.52(0.71)	1.59(0.72)	−2.23	0.026	−0.08	−0.14 to −0.01
CHIA_27	1.48(0.68)	1.53(0.66)	−1.52	0.128	−0.05	−0.11 to 0.01
CHIA_28	1.42(0.70)	1.54(0.71)	−3.68	<0.001	−0.13	−0.19 to −0.06
CHIA_30	2.00(0.90)	2.22(0.92)	−4.98	<0.001	−0.22	−0.30 to −0.13
CHIA_31	2.01(0.87)	2.02(0.82)	−0.22	0.827	−0.01	−0.09 to 0.07
CHIA_32	1.76(0.82)	1.92(0.81)	−4.20	<0.001	−0.16	−0.24 to −0.09
CHIA_33	1.62(0.84)	1.82(0.85)	−4.94	<0.001	−0.20	−0.28 to −0.12

**Items and scales**	**Male(n=753)** ***M(SD)***	**Female(n=1037)** ***M(SD)***	***t***	***P***	**Mean Difference**	**95% Confidence Interval**
CHIA_34	1.54(0.75)	1.66(0.72)	−3.26	<0.001	−0.12	−0.18 to −0.05
CHIA_35	2.48(1.00)	2.22(0.93)	5.53	<0.001	0.26	0.17 to 0.35
CHIA_36	2.75(0.95)	2.80(0.87)	−1.31	0.186	−0.06	−0.14 to 0.03
CHIA_37	2.58(0.97)	2.77(0.87)	−4.21	<0.001	−0.19	−0.27 to −0.10
CHIA_38	1.66(0.81)	1.80(0.84)	−3.55	<0.001	−0.14	−0.22 to −0.06
CHIA_FRUST	19.60(4.92)	20.44(4.42)	−3.72	<0.001	−0.84	−1.28 to −0.40
CHIA_PHYS	22.47(5.75)	23.47(4.95)	−3.82	<0.001	−0.99	−1.50 to −0.48
CHIA_PEER	17.17(4.72)	18.59(4.43)	−6.50	<0.001	−1.42	−1.84 to −0.99
CHIA_AUTH	18.94(5.23)	20.25(4.98)	−5.38	<0.001	−1.31	−1.79 to −0.83
CHIA_Total	78.18(18.05)	82.74(16.31)	−5.49	<0.001	−4.56	−6.19 to −2.93

Note. M, mean; *SD*, standard deviation; FRUST, Frustration subscale; PHYS, Physical Aggression subscale; PEER, Peers Relationships subscale; AUTH, Authority Relations subscale.
